# Structured Motor Play and Preschool Task Performance: An Exploratory Single-Site Randomized Pilot Study in Tunisian Children

**DOI:** 10.3390/children13101377

**Published:** 2026-10-10

**Authors:** Rahma Loussaief, Amayra Tannoubi, Anis Ben Chikha, Chiraz Goumni, John Elvis Hagan Jnr, Medina Srem-Sai, Najha Bouallagui, Vlad Adrian Geantă, Riadh Khalifa

**Affiliations:** 1 Department of Education, Higher Institute of Continuing Education and Training, University of Tunis, Tunis 1007, Tunisia; rahma.loussaief2016@gmail.com; 2 Research Laboratory (LR23JS01) “Sport Performance, Health and Society”, Higher Institute of Sport and Physical Education of Ksar-Said, University of Manouba, Tunis 2010, Tunisia; benchikhaanis@yahoo.fr (A.B.C.); chirazgoumni400@gmail.com (C.G.); boualleguinejha6@gmail.com (N.B.); riadhkhal@yahoo.fr (R.K.); 3 Physical Activity, Sport and Health, Research Unit (UR18JS01), National Observatory of Sport, Tunis 1003, Tunisia; amayra.tannoubi@issepgf.ugaf.tn; 4 High Institute of Sport and Physical Education of Gafsa, University of Gafsa, Gafsa 2100, Tunisia; 5 Sports Performance Optimization Research Laboratory (LR09SEP01), National Center for Sports Medicine and Science (CNMSS), Tunis 1003, Tunisia; 6 The Higher Institute of Sport and Physical Education (Ksar Saïd), University of Manouba, Manouba 2010, Tunisia; 7 Department of Health, Physical Education and Recreation, University of Cape Coast, Cape Coast PMB TF0494, Ghana; 8 Neurocognition and Action—Biomechanics Research Group, Faculty of Psychology and Sports Science, Bielefeld University, 33501 Bielefeld, Germany; 9 Department of Health, Physical Education, Recreation and Sports, University of Education, Winneba P.O. Box 25, Ghana; mssai@uew.edu.gh; 10 Department of Physical Education and Sport, Faculty of Physical Education and Sport, Aurel Vlaicu University of Arad, 310010 Arad, Romania

**Keywords:** structured motor play, preschool education, Stroop interference, response inhibition, immediate affective valence, intervention-aligned mathematics task, Tunisia

## Abstract

**Highlights:**

**What are the main findings?**

- A 12-week structured motor play program was associated with fewer Go-No-Go commission errors and higher immediate affective valence than usual preschool activities, with no between-group difference in derived Stroop interference time.
- The mathematics finding did not remain significant after false-discovery-rate correction and involved intervention-practiced content.

**What are the implications of the main findings?**

- High attendance (93%) and instructor adherence (approximately 95%) suggest that the program was deliverable in this preschool setting.
- Larger, prospectively registered trials with attention-matched controls are needed before conclusions about generalized cognitive, academic, or implementation benefits can be drawn.

**Abstract:**

Background: Cognitively enriched motor play activities may engage movement, rule following, attention, response inhibition, spatial processing, and early mathematical concepts during early childhood. Experimental evidence from preschool settings remains limited, particularly in North African contexts. This study examined short-term between-group differences associated with a structured motor play program in Stroop interference, response inhibition, intervention-aligned mathematics performance, and immediate affective valence among Tunisian preschool children. Methods: This exploratory randomized pilot study included 40 preschool children (mean age = 4.06 years, SD = 0.17) from a single preschool in Tunisia. Children were allocated to either a structured motor play group (*n* = 20) or a usual preschool activities group (*n* = 20). The intervention was delivered twice weekly for 12 weeks. Outcomes were assessed at baseline and immediately post-intervention. Between-group differences in change scores were examined using Welch’s *t*-tests, Mann–Whitney *U* tests, and Hedges’ *g* effect sizes. A Benjamini–Hochberg false-discovery-rate procedure was applied across the four exploratory outcome comparisons. Results: Groups showed similar changes in derived Stroop interference time (−1.00 vs. −0.85 s; *p* = 0.893). Compared with the usual preschool activities group, the structured motor play group demonstrated a greater reduction in Go/No-Go commission errors (−0.55 vs. 0.05; *p* < 0.001; Hedges’ *g* = −1.49) and a greater increase in immediate affective valence (1.45 vs. 0.05; *p* < 0.001; Hedges’ *g* = 2.12). The between-group difference in intervention-aligned mathematics task change was marginally significant before adjustment (*p* = 0.048) but was not retained after false-discovery-rate correction (*q* = 0.065). Conclusions: A 12-week structured motor play program was associated with improved performance on a preschool Go/No-Go response inhibition task and more positive immediate affective valence, whereas no between-group difference was observed in derived Stroop interference time. The mathematics finding was not retained after adjustment for multiple comparisons. These preliminary findings support further research on structured motor play approaches as a potentially valuable component of early-childhood education. Trial Registration: Open Science Framework, 10.17605/OSF.IO/ZTKV4; registered retrospectively on 6 March 2026, after completion of recruitment, intervention delivery, and post-intervention assessment.

## 1. Introduction

The preschool period represents a foundational phase of human development during which cognitive, motor, emotional, and social capacities undergo rapid and closely interconnected changes [1]. Among the most consequential domains emerging during this period are executive functions, including inhibitory control, working memory, and cognitive flexibility, which support children’s ability to regulate behavior, sustain attention, follow rules, and adapt to changing task demands [2,3,4,5]. At the same time, early mathematical abilities including number sense, spatial reasoning, classification, and pattern recognition begin to emerge, providing the conceptual basis for formal mathematical learning [6,7].

Increasing evidence suggests that motor and cognitive development are closely interconnected in early childhood [8,9,10]. This view is consistent with embodied cognition, which emphasizes the role of bodily interaction with the environment in cognitive development [11,12]. Sensorimotor experiences may link perception, action, and higher-order cognitive processes [13]. Accordingly, fine and gross motor skills have been associated with executive functions, attentional control, and early numeracy, while stronger motor competence has also been related to later academic performance in mathematics [1,14,15,16,17].

Play constitutes a central mechanism through which young children explore their environment and develop cognitive and socio-emotional competencies [18]. Play-based learning is therefore widely recognized as an important component of early childhood education [19,20]. Motor play encompasses a wide range of activities that activate the sensorimotor system, including running, climbing, throwing, balancing, and object manipulation [21,22]. When such activities are deliberately organized around rules, goals, sequencing, or cognitive challenges, they may also engage planning, inhibition, attention, and decision-making [23,24,25]. Recent quasi-experimental evidence indicates that a narrative-based outdoor motor intervention produced greater motor-competence gains than a comparison condition, whereas inhibitory control improved over time in both groups without a significant between-group difference [26]. This distinction suggests that motor and inhibitory control outcomes may respond differently to movement-based interventions and does not indicate that the narrative intervention improved inhibitory control specifically.

Neuroscientific research offers biological evidence supporting the relationship between motor activity and cognitive development [25,27]. Shared neural circuits involving the prefrontal cortex, cerebellum, and parietal regions are engaged during both motor planning and higher-order cognitive tasks [28,29]. These neural networks are critically involved in executive functions such as inhibitory control, working memory, and cognitive flexibility [30,31]. The overlap between motor and cognitive neural systems may help explain why movement-based interventions have the potential to enhance both executive functioning and academic outcomes in young children [32,33].

Despite the recognized developmental importance of movement-based learning, the extent to which structured motor play is integrated into preschool curricula, in Tunisia or internationally, has not been systematically documented and is not established by the intervention-effect literature cited above. We are not aware of curriculum-prevalence data for Tunisian preschool settings, and this manuscript does not claim to establish such prevalence; the present study instead examines the effects of one structured program relative to usual preschool activities in a single setting.

Affective valence represents another potentially important dimension of movement-based learning. Early self-regulation, attention, motivation, and task persistence are closely related to children’s educational experiences [34,35,36]. Positive affective experiences during play can facilitate cognitive processing, enhance memory retention, and support sustained engagement with learning tasks [37,38]. Assessing affective valence within educational interventions therefore provides valuable insight into how movement-based learning experiences influence both cognitive and emotional aspects of development.

Despite these converging perspectives, important gaps remain. Much of the evidence linking motor skills, executive functions, and academic performance is based on observational, correlational, or longitudinal designs [34,35]. Systematic reviews indicate a growing body of intervention research targeting executive function, self-regulation, motor skills, physical activity, and early academic outcomes in preschool children; however, findings remain heterogeneous across intervention approaches, dose, delivery agents, outcome measures, study quality, and developmental domains [39,40].

These gaps highlight the need for cautiously interpreted exploratory studies examining associations between structured motor play programs and developmental outcomes in preschool settings. The present program combined motor activities with rule following, response inhibition, attentional demands, spatial processing, sequencing, counting, shape recognition, and classification. This study aimed to estimate short-term between-group differences in a derived Stroop interference-time index, a Go/No-Go response inhibition task, a brief researcher-developed mathematics task aligned with intervention content, and immediate self-reported affective valence. Given the small sample, retrospective registration, single-site design, usual-curriculum comparator, and immediate post-intervention assessment, the study was intended to provide preliminary estimates for future confirmatory research rather than definitive evidence of efficacy.

## 2. Materials and Methods

### 2.1. Research Design

The study was registered retrospectively with the Open Science Framework (OSF; 10.17605/OSF.IO/ZTKV4) on 6 March 2026, after recruitment, intervention delivery, and post-intervention assessments had been completed. No publicly accessible protocol or statistical analysis plan was time-stamped before study commencement; consequently, the prospectively intended primary-versus-secondary outcome hierarchy, multiplicity plan, and trial-success criterion cannot be independently verified, and selective reporting cannot be excluded. Accordingly, the four outcome domains reported in this manuscript—derived Stroop interference time, Go/No-Go commission errors, intervention-aligned mathematics task score, and immediate affective valence—are treated as exploratory outcomes, and all analyses and findings should be interpreted accordingly. The Benjamini–Hochberg false-discovery-rate procedure was applied post hoc across the four exploratory between-group change score comparisons to provide a transparent multiplicity-adjusted sensitivity assessment; it was not a prospectively specified trial-success criterion. A transparency classification of study outcomes and analyses is provided in Supplementary Table S5.

**Figure 1:**
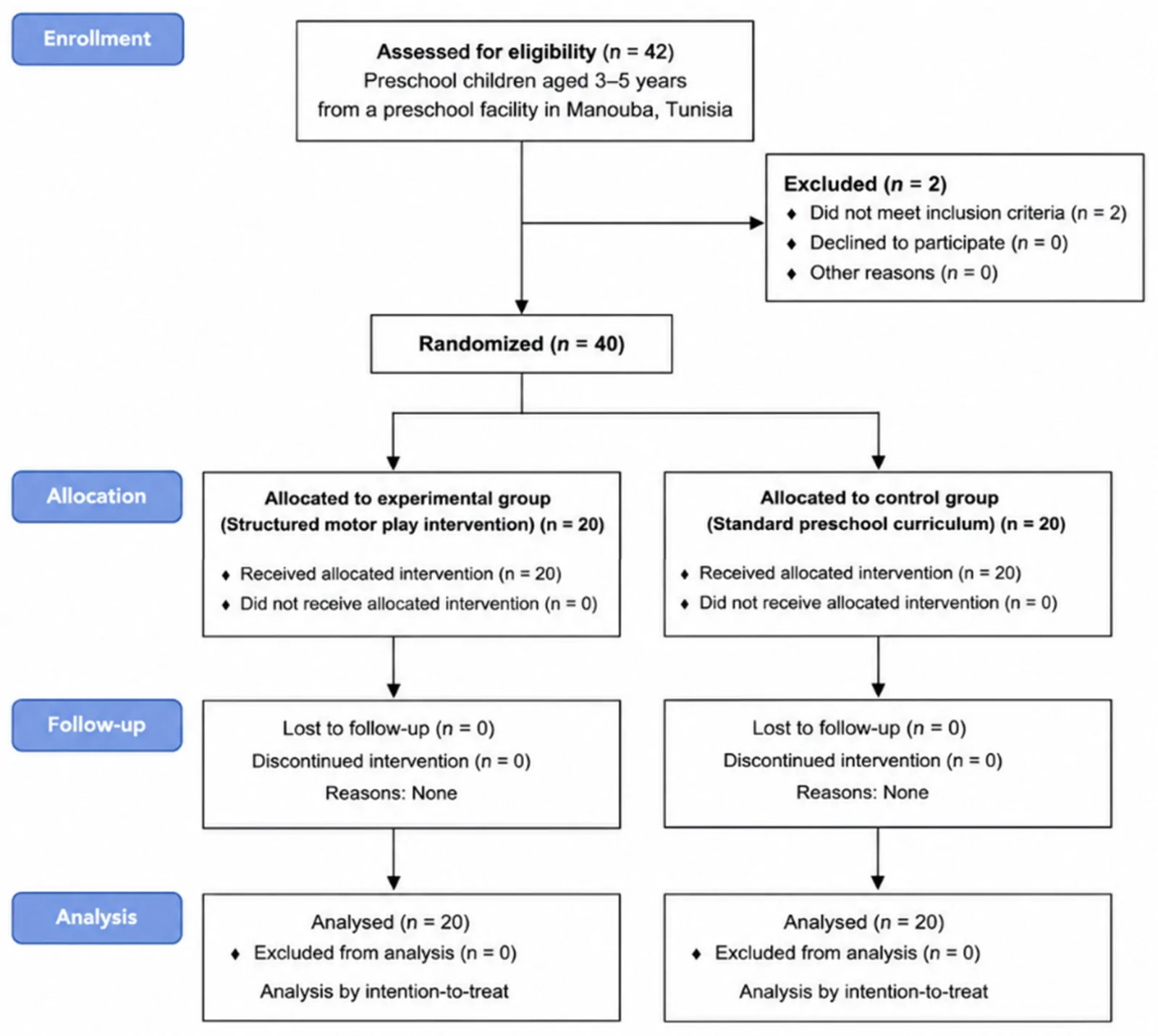
Flow diagram illustrating participant recruitment, allocation, and follow-up.

A computer-generated 1:1 allocation sequence was prepared before participant enrolment by an independent researcher who was not involved in participant recruitment, intervention delivery, or outcome assessment. Simple randomization was used, with each child having an equal probability of allocation to the structured motor play group or the usual preschool activities group. The random sequence was generated using https://www.randomizer.org/. Allocation assignments were placed in sequentially numbered, opaque, sealed envelopes before enrolment. The envelopes were prepared by the independent researcher, stored securely by a member of the research team not involved in outcome assessment, and opened in numerical order by the study coordinator after completion of baseline assessment and confirmation of eligibility. The researcher who generated the allocation sequence did not participate in enrolment or assignment. The study coordinator, who enrolled participants and opened envelopes, had access only to the next sequential envelope at the time of assignment and did not have access to the full allocation sequence in advance (see Figure 1).

Blinding of children, parents or legal guardians, preschool staff, and intervention instructors was not feasible because of the nature of the intervention. Outcome assessors were not blinded to group allocation because of staffing and logistical constraints. Data analysis was undertaken by a member of the research team who was not involved in intervention delivery and was not masked to group allocation. No formal assessment of blinding success was conducted. The absence of outcome assessor and analyst blinding represents a potential source of detection and analytical bias and should be considered when interpreting the findings.

### 2.2. Participants

An a priori sample-size calculation was based on an independent-samples, two-sided *t*-test with *α* = 0.05 and target power of 80%. The original calculation assumed a large standardized mean difference (*d* = 0.80). Under this assumption, approximately 26 children per group, or 52 children in total, would be required. Under more conservative assumptions, approximately 45 children per group would be required to detect *d* = 0.60, and approximately 100 children per group would be required to detect *d* = 0.40. The final sample of 40 children, with 20 children per group, was therefore underpowered for small-to-moderate effects and did not achieve 80% power under the original large-effect assumption. The present study should consequently be interpreted as an exploratory pilot study intended to provide preliminary estimates for a future adequately powered trial.

Participants were recruited from a preschool facility located in Manouba, Tunisia. Initially, 42 children aged between 3 and 5 years were screened for eligibility. Following the screening process, two children did not meet the inclusion criteria and were therefore excluded. The final sample consisted of 40 preschool children, who were randomly allocated to either the experimental group (*n* = 20) or the control group (*n* = 20). Participant recruitment and baseline assessments were conducted between September and October 2025, while the intervention and post-intervention assessments were completed between October 2025 and January 2026.

Eligibility criteria required that children attend preschool regularly, present no diagnosed neurodevelopmental disorders, and have parental consent for participation in the study. Children were excluded if they had previously participated in structured physical activity interventions or if they had medical conditions that could contraindicate participation in moderate physical activity. No adverse events, injuries, or unintended effects related to the intervention were observed or reported during the study period.

Ethical approval for the study was granted by the Research Ethics Committee of the Higher Institute of Sport and Physical Education of Ksar-Saïd (CPP N°19/2025, dated 15 March 2025). All procedures were conducted in accordance with the ethical principles outlined in the Declaration of Helsinki [41]. Written informed consent and verbal assent were obtained from the parents or legal guardians of all participants prior to their inclusion in the study.

### 2.3. Intervention Protocol

Children assigned to the experimental group participated in a structured motor play intervention implemented over a 12-week period (Table 1), with two sessions per week lasting approximately 45 min each. The session structure incorporated a warm-up phase, a series of structured motor play tasks integrating cognitive and mathematical components, a short game-based challenge, and a brief cool-down period. The general organization of each intervention session is presented in Table 2. The program was designed to provide developmentally appropriate stimulation of both gross and fine motor skills through a variety of structured play-based activities. Sessions incorporated tasks such as obstacle navigation, ball manipulation and throwing exercises, jumping and balance games, shape-sorting activities, and cooperative challenges aimed at promoting coordinated motor engagement.

The intervention followed a progressive structure in which task difficulty increased gradually across sessions to match children’s developmental capacities and maintain engagement. In addition to the motor demands, several activities incorporated cognitive components intended to stimulate executive functioning processes. These elements included rule adherence, response inhibition, simple decision-making, and attentional regulation embedded within play scenarios. All sessions were delivered by trained physical education specialists who followed a standardized intervention protocol to ensure consistency in implementation across the intervention period.

Children assigned to the usual preschool activities group continued their regular preschool routine. This included classroom activities, free play, teacher-guided learning activities, coloring, letter and number recitation, and routine outdoor recess. The comparison condition was not designed to match the structured motor play program in adult contact, session structure, novelty, social interaction, expectancy, or time spent in organized activities. Therefore, any observed between-group differences cannot be attributed specifically to motor play and may partly reflect nonspecific effects associated with additional adult attention, structured participation, novelty, or group interaction.

Attendance, number of sessions delivered, and protocol deviations were recorded throughout the intervention period. A total of 24 of 24 planned sessions were delivered over the 12-week intervention period, corresponding to two approximately 45 min sessions per week. Mean participant attendance was 93% (range: 85–100%).

Fidelity to the standardized intervention protocol was assessed through video review by trained research personnel, with a mean adherence rate of approximately 95%. No major protocol deviations were identified during the intervention period. These attendance and adherence figures are descriptive delivery indicators recorded during the study and are distinct from a formal implementation evaluation, which would additionally require a predefined coding rubric, multiple independent coders, and inter-rater agreement statistics. These process indicators describe intervention delivery in the present preschool only and should not be interpreted as evidence of broader feasibility, scalability, cost-effectiveness, or acceptability. The study did not formally assess implementation costs, resource requirements, instructor training duration, teacher workload, teacher adherence, parental acceptability, school acceptability, or implementation across multiple preschool settings.

Additional information on intervention delivery, attendance, and available fidelity indicators is provided in Supplementary Table S4.

### 2.4. Outcome Measures

#### 2.4.1. Stroop Task Performance

The Victoria version of the Stroop Color–Word Test [42] was administered individually in Tunisian Arabic by trained research personnel using standardized instructions. The assessment comprised three card conditions: Card 1 (colored dots); Card 2 (neutral words printed in incongruent ink colors); and Card 3 (color words printed in incongruent ink colors).

Completion time was recorded in seconds from presentation of each card until the child completed the required response. The primary Stroop outcome was derived interference time, calculated as Card 3 completion time minus Card 1 completion time:

Derived interference time = Card 3 completion time − Card 1 completion time.

Larger values indicate a greater additional time cost for the Card 3 condition relative to Card 1, whereas lower values indicate lower derived interference time. For an incorrect response, the examiner instructed the child to continue without correction. Omissions, nonresponses, and incomplete card administrations were retained in the dataset rather than excluded. Instructions could be repeated once when necessary to ensure task comprehension, consistent with the repetition procedure used for the other outcome measures in this study; no corrective feedback was provided once a score card had begun. Completion times were recorded according to a prespecified timing rule described above. No incomplete card administrations occurred in this sample; all 40 children completed all three cards at both assessment occasions, so the derived interference-time formula was calculated from full completion times in every case.

The psychometric properties of this exact Stroop administration procedure have not been independently established in Tunisian preschool children. Therefore, derived interference time should be interpreted as performance on the specific Stroop-derived index used in this study rather than as a comprehensive measure of executive functioning or generalized interference control.

#### 2.4.2. Go/No-Go Task

Motor-response inhibition was assessed using a simplified Go/No-Go task adapted from the tapping subtest of the Frontal Assessment Battery, modified for preschool-aged children [43]. Children were instructed to respond when the examiner produced a single tap and to withhold their response when the examiner produced a double tap. The task was administered individually in Arabic by trained research personnel following standardized instructions.

Children completed two practice trials followed by six test trials, comprising three Go trials and three No-Go trials presented in a fixed alternating sequence (Go, No-Go, Go, No-Go, Go, No-Go). The commission-error score ranged from 0 to 3, corresponding to the maximum possible number of No-Go trials. The primary outcome was the number of commission errors, defined as responses made during No-Go trials in which inhibition was required. Higher commission-error values indicated poorer performance on the administered response inhibition task.

Omissions and unclear responses were scored as correct inhibition and were not counted as commission errors. Requests for repeated instructions were permitted once during the practice phase. The Frontal Assessment Battery is cited here only as the source of the tapping-task adaptation and not as evidence of psychometric validity in this population. The psychometric properties of this adapted Go/No-Go procedure have not been independently established in Tunisian preschool children. Accordingly, the results should be interpreted as performance on the administered response inhibition task rather than as definitive evidence of generalized inhibitory control ability.

#### 2.4.3. Brief Researcher-Developed Mathematics Task

Early mathematics performance was assessed using a brief, researcher-developed, three-item task targeting the foundational mathematical concepts practiced during the intervention. The task was administered individually at baseline and immediately post-intervention, in a quiet preschool area, using standardized verbal instructions and identical materials on both occasions (approximately 3–5 min per child).

Three items were assessed: (1) identification of a basic shape (circle or square), (2) counting a set of one to five objects, and (3) classification by color or size. Children responded verbally and/or by pointing. Each correct response received one point (incorrect/nonresponses received zero), yielding a total score of 0–3. Instructions could be repeated once for comprehension; no corrective feedback, hints, or teaching were given during assessment.

Before the main study, in a six-child feasibility pilot, internal consistency was α = 0.85, and the test–retest correlation was r = 0.72. Because these estimates were based on a very small pilot sample, they are unstable and should not be interpreted as evidence of formal psychometric validation.

Given the discrete 0–3 scoring and direct overlap between assessed and practiced content, the mathematics score was interpreted cautiously. The primary analysis compared change scores between groups, with a Mann–Whitney U test as a nonparametric sensitivity analysis.

#### 2.4.4. Immediate Affective Valence

Immediate affective valence was assessed using the Feeling Scale, a single-item, 11-point bipolar rating scale ranging from −5 (“very bad”) to +5 (“very good”) [44]. The scale was administered individually at each assessment occasion in a quiet area of the preschool setting by trained research staff. To facilitate comprehension among preschool-aged children, the Feeling Scale was presented on a large horizontal visual display containing the numerical response options from −5 to +5 and age-appropriate facial-expression symbols. The display represented a continuum from a very sad face at −5, through a neutral face at 0, to a very happy face at +5.

The assessment was explained to children in age-appropriate Tunisian Arabic using standardized instructions: “How do you feel right now? You can show me if you feel very bad, bad, neither bad nor good, good, or very good.” Children were asked to indicate the position on the visual scale that best represented how they felt at that moment by pointing to the corresponding number or facial-expression symbol. Instructions could be repeated once when necessary to ensure comprehension of the response format; no suggestions, feedback, or guidance regarding the child’s response were provided.

Higher positive scores represented more pleasant immediate affective states, scores close to zero represented neutral affective states, and lower negative scores represented more unpleasant immediate affective states. The Feeling Scale was used as an indicator of children’s self-reported affective valence at the time of assessment. As the measure comprises a single item, internal consistency indicators such as Cronbach’s alpha were not applicable.

Before the main study, the administration procedure and visual response display were pilot tested with six Tunisian preschool children of comparable age. The pilot phase supported the clarity, feasibility, and age-appropriateness of the Tunisian Arabic instructions, facial-expression symbols, and child-response procedure. Feeling Scale scores were analyzed as an indicator of immediate affective valence at each assessment occasion.

The pilot procedure addressed feasibility and child comprehension only; it did not establish construct validity, responsiveness to change, test–retest reliability, or psychometric equivalence of the Feeling Scale administration procedure in Tunisian preschool children.

Detailed information on the available administration, scoring, and measurement documentation for all study outcomes is provided in Supplementary Table S1.

### 2.5. Statistical Analyses

Data were examined for completeness, data-entry errors, outliers, and distributional characteristics. No missing outcome data were observed among the 40 randomized children. Descriptive statistics are presented as means and standard deviations for baseline, post-intervention, and individual change scores.

The primary analyses were exploratory between-group comparisons of individual pre-to-post change scores (*T*2 − *T*1). Welch’s independent-samples *t*-tests were used to compare mean change scores because equality of variances was not assumed. Hedges’ *g*, with 95% bootstrap confidence intervals based on 2000 resamples, was calculated as an exploratory standardized estimate of the between-group difference in change [45].

Mann–Whitney *U* tests were conducted as nonparametric sensitivity analyses, particularly for bounded, discrete, or non-normally distributed outcomes. The Benjamini–Hochberg false-discovery-rate procedure was applied across the four primary between-group change score comparisons [46]. Additional bounded-outcome sensitivity analyses for the mathematics task are reported in Supplementary Table S3.

Because the mathematics outcome was bounded, discrete, and restricted to four possible values, an exact two-sided permutation test comparing the between-group difference in mean change scores was conducted as an additional post hoc sensitivity analysis. The observed test statistic was the absolute difference in mean change scores between groups. The permutation analysis was based on all possible reallocations of 20 participants to each group under equal allocation. This analysis was conducted as a sensitivity analysis and was not included in the false-discovery-rate multiplicity family.

All tests were two-sided, with the significance level set at *α* = 0.05. Given the small sample, retrospective registration, multiple outcomes, and exploratory study aim, *p*-values and effect sizes are interpreted as descriptive estimates rather than confirmatory evidence. Analyses were performed using IBM SPSS Statistics Version 27 (IBM Corp., Armonk, NY, USA), and figures were generated in RStudio, version 4.4.0 (R Foundation for Statistical Computing, Vienna, Austria), within RStudio (Posit Software, PBC, Boston, MA, USA), using ggplot2 and dplyr [47,48]. See Supplementary Table S5 (Section 2.1) for the full classification of outcomes and analyses referenced above.

## 3. Results

### 3.1. Baseline Characteristics and Descriptive Statistics

A total of 40 preschool children completed both the baseline and immediate post-intervention assessments, with *n* = 20 in each group. Table 3 provides a descriptive summary of participant characteristics. Because the sample was small, any non-significant baseline differences should not be taken as proof that the groups were comparable across all measured or unmeasured attributes. Table 4 presents the baseline and post-intervention descriptive statistics, along with summaries of each participant’s change scores.

The distribution of mathematics task scores by group and assessment occasion is presented in Supplementary Table S2. No child received a score of 0 at either assessment. At baseline, 7 children (35.0%) in the structured motor play group and 10 children (50.0%) in the usual preschool activities group scored 1. Immediately after the intervention, 9 children (45.0%) in the structured motor play group and 4 children (20.0%) in the usual preschool activities group scored 3. Taken together, these frequencies demonstrate the limited, discrete 0–3 range of the mathematics outcome and justify a cautious reading of analyses that handle this outcome as if it were continuous.

Descriptive statistics for both groups at baseline (*T*1) and post-intervention (*T*2), together with change scores (*T*2 − *T*1), are presented in Table 4.

Descriptive statistics for baseline, immediate post-intervention, and individual change scores are presented in Table 4. For derived Stroop interference time, the structured motor play group decreased from 32.20 s (*SD* = 3.30) at baseline to 31.20 s (*SD* = 5.28) at immediate post-intervention, whereas the usual preschool activities group decreased from 32.85 s (*SD* = 3.27) to 32.00 s (*SD* = 3.20). Thus, mean change in derived interference time was similar between groups (−1.00 vs. −0.85 s).

**Figure 2:**
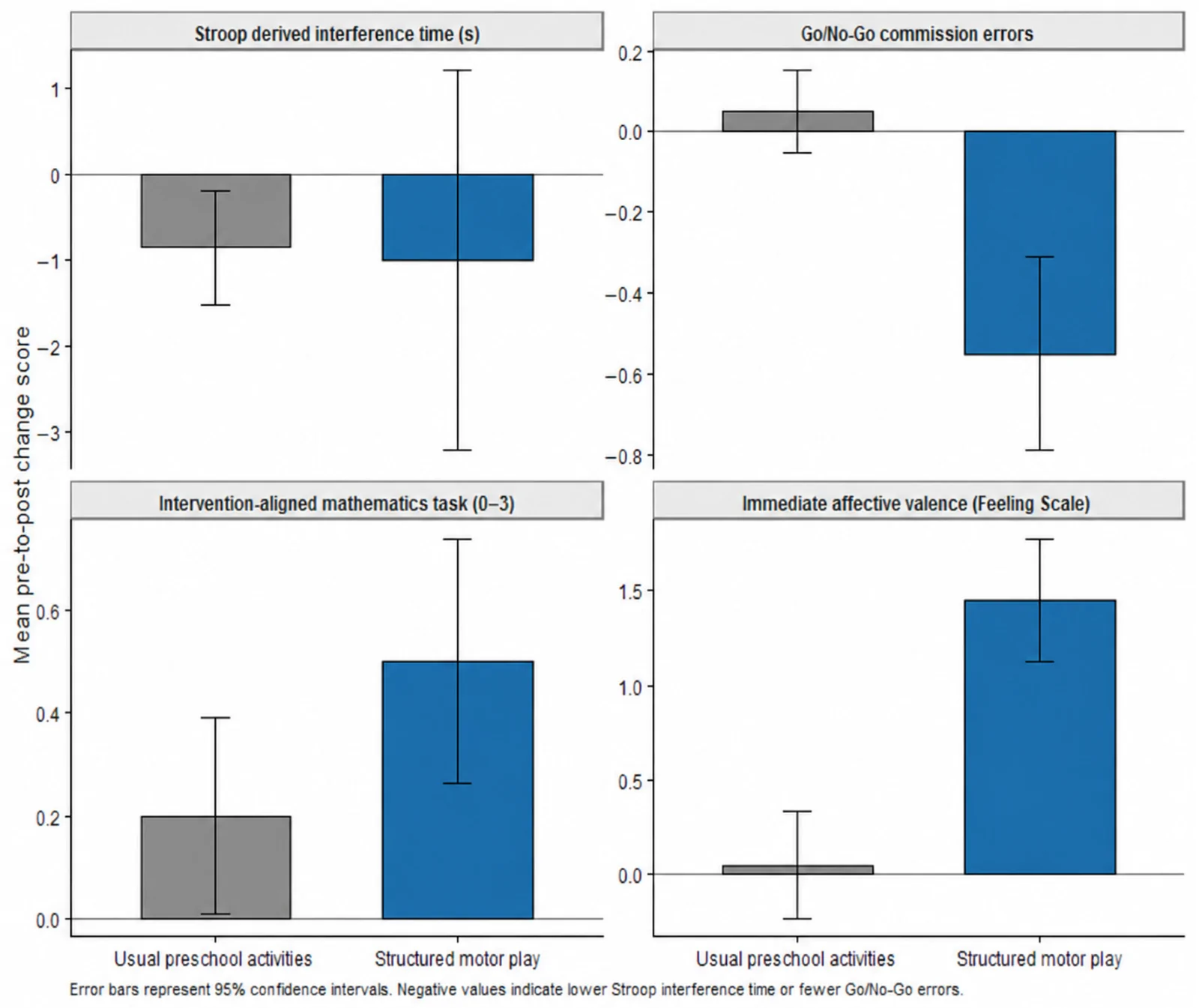
Exploratory group differences in pre-to-post change scores. Bars represent mean pre-to-post change scores, and error bars represent 95% confidence intervals. Negative values indicate lower derived Stroop interference time or fewer Go/No-Go commission errors at immediate post-intervention. Positive values indicate higher intervention-aligned mathematics task scores and more positive immediate affective valence. The figure presents unadjusted descriptive change scores.

Go/No-Go commission errors decreased from 2.15 (*SD* = 0.75) to 1.60 (*SD* = 0.60) in the structured motor play group and increased from 2.25 (*SD* = 0.55) to 2.30 (*SD* = 0.57) in the usual preschool activities group. Scores on the brief intervention-aligned mathematics task increased from 1.90 (*SD* = 0.79) to 2.40 (*SD* = 0.60) in the structured motor play group and from 1.60 (*SD* = 0.68) to 1.80 (*SD* = 0.77) in the usual preschool activities group. Immediate affective valence scores increased from 2.40 (*SD* = 0.60) to 3.85 (*SD* = 0.67) in the structured motor play group and from 3.65 (*SD* = 0.49) to 3.70 (*SD* = 0.57) in the usual preschool activities group. Group differences in change scores are illustrated in Figure 2.

### 3.2. Effects of the Motor Play Intervention

Exploratory between-group comparisons of individual pre-to-post change scores are presented in Table 5. The primary analyses used Welch’s independent-samples *t*-tests. Mann–Whitney *U*tests were conducted as nonparametric sensitivity analyses, and false-discovery-rate adjustment was applied across the four primary outcome comparisons.

The exact two-sided permutation sensitivity analysis also did not indicate a statistically significant between-group difference in mathematics task change scores (*p* = 0.096; Supplementary Table S3). Thus, the mathematics finding was not robust across the unadjusted Welch comparison, false-discovery-rate-adjusted analysis, Mann–Whitney sensitivity analysis, and exact permutation sensitivity analysis.

For derived Stroop interference time, the structured motor play group and the usual preschool activities group showed similar mean reductions from baseline to immediate post-intervention (−1.00 vs. −0.85 s), Welch’s *t*(22.41) = −0.14, *p* = 0.893, FDR-adjusted *q* = 0.893. The nonparametric sensitivity analysis was also not statistically significant (*U* = 260.5, *p* = 0.098). The exploratory standardized difference was negligible (*g* = −0.04, 95% bootstrap CI [−0.56, 0.80]). Therefore, the study did not provide evidence of a short-term between-group difference in the derived Stroop interference-time score.

For Go/No-Go commission errors, the structured motor play group showed a larger mean reduction than the usual preschool activities group (−0.55 vs. 0.05 errors), Welch’s *t*(26.03) = −4.82, *p* < 0.001, FDR-adjusted *q* < 0.001. The Mann–Whitney sensitivity analysis was statistically significant (*U* = 85.5, *p* < 0.0001). The exploratory standardized difference was large (*g* = −1.49, 95% bootstrap CI [−2.34, −0.99]). This result indicates a short-term between-group difference in performance on the administered response inhibition task; it should not be interpreted as definitive evidence of generalized inhibitory control improvement.

For the brief intervention-aligned mathematics task, the structured motor play group showed a larger mean score increase than the usual preschool activities group (0.50 vs. 0.20 points), Welch’s *t*(36.25) = 2.04, unadjusted *p* = 0.048, *g* = 0.63, 95% bootstrap CI [0.00, 1.45]. However, the result did not remain statistically significant after false-discovery-rate adjustment (*q* = 0.065), and the Mann–Whitney sensitivity analysis was marginal (*U* = 260.0, *p* = 0.051). Given the restricted 0–3 score range, direct overlap between outcome content and intervention activities, and inconsistency across inferential approaches, this finding should be regarded as inconclusive exploratory evidence regarding intervention-aligned trained-task performance. It does not support inferences about generalized mathematics ability, numeracy, or school readiness.

Immediate self-reported affective valence increased more in the structured motor play group than in the usual preschool activities group (mean change: 1.45 vs. 0.05 points), Welch’s *t*(37.41) = 6.84, *p* < 0.001, FDR-adjusted *q* < 0.001. The Mann–Whitney sensitivity analysis was statistically significant (*U* = 374.0, *p* < 0.001). The exploratory standardized difference was large (*g* = 2.12, 95% bootstrap CI [1.55, 3.23]). This result concerns a single immediate affective valence rating and does not establish broader changes in emotional well-being, sustained affective functioning, classroom engagement, or motivation. Because the groups differed at baseline on this outcome (structured motor play: M = 2.40; usual preschool activities: M = 3.65), the large standardized change score effect reflects greater room for improvement in the structured motor play group rather than a correspondingly large separation in final scores (M = 3.85 vs. 3.70 at immediate post-intervention). As a sensitivity check, a baseline-adjusted analysis of covariance was conducted for this outcome, since the group × baseline interaction was non-significant (*p* = 0.883), indicating the homogeneity-of-regression-slopes assumption was met for this outcome only. The baseline-adjusted group effect remained statistically significant, F(1,37) = 6.33, *p* = 0.016, with adjusted means of 4.13 (structured motor play) and 3.42 (usual preschool activities) at the grand-mean baseline value, supporting the change score finding while accounting for baseline imbalance.

**Figure 3:**
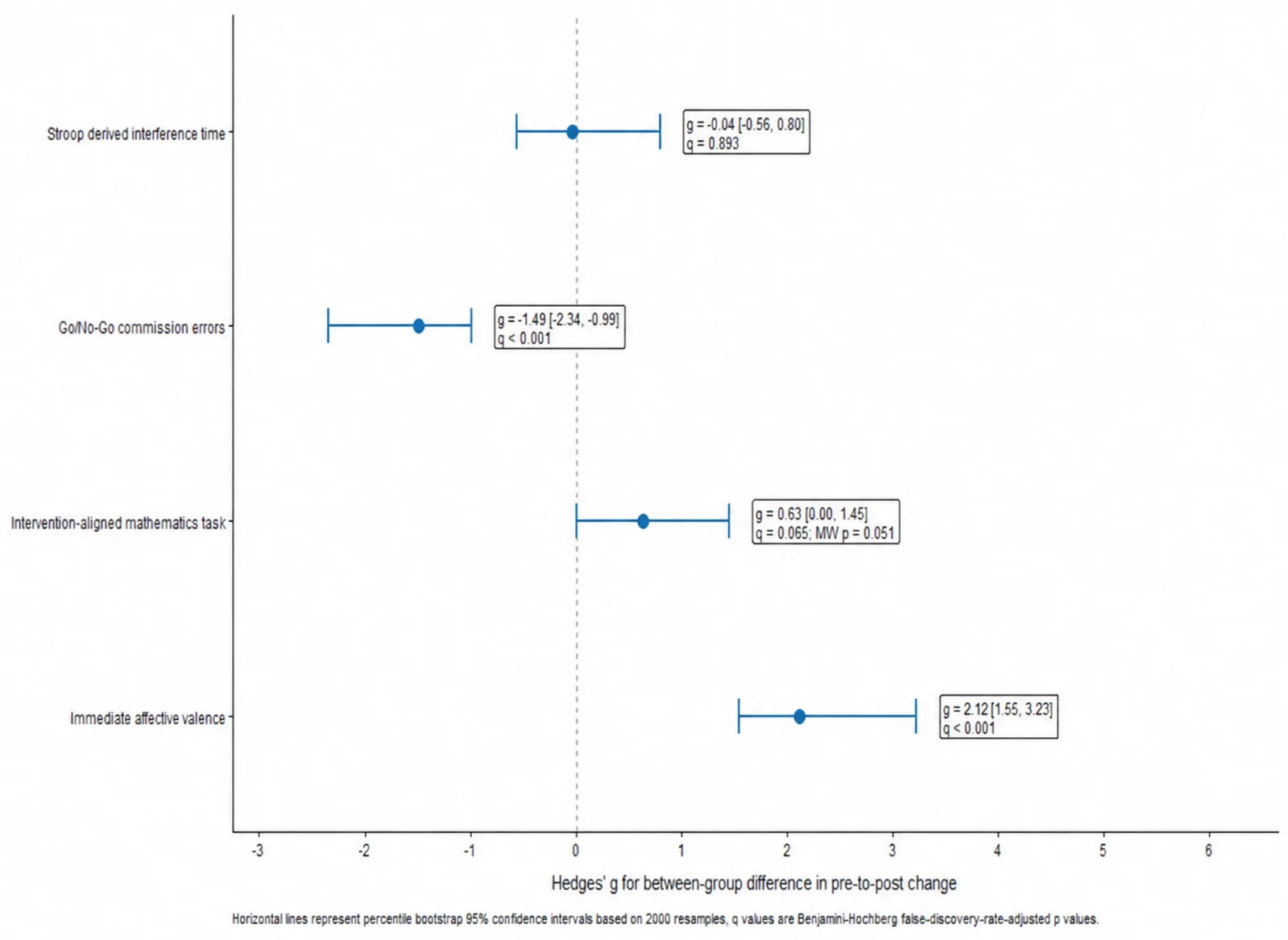
Exploratory standardized between-group differences in pre-to-post change scores. Points represent Hedges’ *g*, and horizontal lines represent percentile bootstrap 95% confidence intervals based on 2000 resamples. Labels report Hedges’ *g*, 95% confidence intervals, and Benjamini–Hochberg false-discovery-rate-adjusted *q*-values. For the intervention-aligned mathematics task, the label also reports the Mann–Whitney sensitivity-analysis result. Negative values indicate larger reductions in derived Stroop interference time or Go/No-Go commission errors in the structured motor play group.

A graphical summary of the standardized between-group effects is presented in Figure 3.

## 4. Discussion

In this exploratory randomized pilot trial, children allocated to a 12-week structured motor play program showed larger short-term reductions in Go/No-Go commission errors and greater increases in immediate affective valence than children continuing usual preschool activities. No between-group difference was observed in derived Stroop interference time. The mathematics finding was not statistically significant after false-discovery-rate correction, was marginal in the nonparametric sensitivity analysis, and concerned content directly practiced during the intervention. Given the retrospective registration, small sample, and usual-activities comparison condition, these results should be interpreted as preliminary, task-specific estimates rather than definitive evidence that structured motor play improves generalized executive function, broad mathematics ability, school readiness, or sustained affective functioning. These findings are broadly consistent with the embodied-cognition perspective, which proposes that sensorimotor experiences can scaffold higher-order cognitive processes through repeated coupling of action, perception, and rule-guided behavior [40,49]. The executive function and inhibitory control findings are directionally consistent with previous work suggesting that movement contexts embedding stopping, monitoring, sequencing, and rapid rule shifts may support self-regulatory processes in young children. In the present intervention, children were repeatedly required to coordinate movement, follow instructions, inhibit incorrect responses, and adapt behavior across changing play demands, all of which plausibly engage executive-control processes described in developmental and neurocognitive models. These findings also align with prior work indicating that cognitively enriched physical activity may support executive functioning more effectively than movement alone, particularly when tasks require simultaneous motor coordination and cognitive control [4,24].

The absence of a between-group difference in derived Stroop interference time does not support an inference that the intervention improved Stroop-measured interference control. The larger reduction in Go/No-Go commission errors is directionally consistent with the possibility that rule-guided movement activities may engage behavioral control, response monitoring, and attention. However, the present design cannot identify a motor play-specific mechanism [26,29]. The intervention also involved additional adult contact, novelty, structured participation, social interaction, cognitive rules, and repeated task engagement. These factors, alone or in combination, may have contributed to the observed group difference in Go/No-Go task performance [25,50]. In addition, the psychometric properties of the exact preschool task adaptations were not independently established in the target population.

The structured motor play group also reported a larger increase in immediate affective valence than the usual preschool activities group. This pattern may reflect enjoyment of movement, social interaction, novelty, perceived competence, or participation in organized group activities. However, the Feeling Scale was a single immediate rating and does not establish sustained changes in affect, emotional well-being, motivation, or classroom engagement. Interpretation is further limited by the higher baseline Feeling Scale score in the usual preschool activities group, which may have reduced the scope for observable improvement because the scale has an upper boundary of +5.

The mathematics outcome requires particularly cautious interpretation. Shape identification, counting from one to five, and classification were directly embedded in the structured motor play sessions and were also the three components assessed by the outcome measure [1,7,14]. The observed pattern is therefore compatible with practice effects, familiarity, or learning of trained content. It does not demonstrate transfer to untrained mathematics tasks, generalized numeracy, spatial reasoning, or school readiness [51]. Furthermore, the restricted 0–3 score range reduces measurement precision and may increase ceiling effects. Future studies should include independent standardized mathematics assessments containing content not directly practiced during the intervention.

The observed group differences should not be interpreted as evidence that usual preschool instruction is inadequate or that structured motor play is more developmentally appropriate than alternative educational approaches. The comparison condition was not matched for adult attention, novelty, social interaction, expectancy, cognitive stimulation, or structured participation. Therefore, the observed differences may reflect the multicomponent intervention package rather than a motor play-specific effect.

The present study may inform the design of future preschool intervention research but does not provide sufficient evidence for policy-level or routine implementation recommendations. Although the program was delivered within one preschool, the study did not evaluate intervention costs, material requirements, instructor training duration, teacher workload, parent or school acceptability, or scalability across educational settings. Future adequately powered trials should determine whether the intervention produces benefits beyond those associated with attention-matched non-motor activities and whether it can be delivered safely, acceptably, and equitably across diverse preschool contexts.

### 4.1. Limitations

Several limitations constrain the interpretation of these findings. First, the trial was registered only after recruitment, intervention delivery, and outcome assessment had been completed. The study therefore did not have a prospectively accessible protocol or statistical analysis plan, and selective reporting cannot be excluded. Second, the sample was small (*N* = 40) and recruited from a single preschool. The study was underpowered for small-to-moderate effects and did not achieve 80% power under the original large-effect assumption. Therefore, null findings may reflect limited statistical sensitivity, whereas statistically significant effect size estimates may be exaggerated or unstable. Third, the usual preschool activities comparison group was not matched for adult attention, session structure, novelty, social interaction, expectancy, or time in organized activities. The observed between-group differences cannot therefore be attributed specifically to motor play. Fourth, several potentially important variables were not measured, including baseline motor competence, socioeconomic circumstances, sleep quality, nutritional status, habitual physical activity, and other developmental influences. In a small sample, random allocation does not ensure balance on these unmeasured variables. Fifth, the executive function and response inhibition tasks were adapted for preschool children, but the psychometric properties of the exact task versions and administration procedures were not independently established in Tunisian preschool children. The findings should therefore be interpreted as performance on the specific tasks administered rather than as comprehensive estimates of executive functioning or inhibitory control. Sixth, the mathematics outcome contained only three items, had a restricted 0–3 score range, and directly assessed content practiced during intervention sessions. It therefore represents trained-task performance and cannot demonstrate generalized mathematics learning, numeracy transfer, or school readiness. Seventh, the Feeling Scale was a single-item measure of immediate affective valence. It does not establish sustained emotional, motivational, or classroom engagement effects. The baseline difference between groups on this bounded scale further limits interpretation. A baseline-adjusted sensitivity analysis supported the same direction and statistical significance of this effect, but the modest separation in final scores relative to the large standardized change score should temper interpretation of its practical magnitude. Eighth, all outcomes were assessed immediately after the intervention. The study therefore provides no evidence regarding maintenance of the observed differences, later developmental relevance, transition to school, or longer-term academic outcomes. Finally, attendance, intervention fidelity, instructor adherence, teacher workload, training requirements, costs, resource use, parental acceptability, school acceptability, and scalability were not formally evaluated. The intervention also combined multiple components, including gross motor activity, fine motor activity, cognitive rules, mathematical content, adult scaffolding, and social interaction. The design cannot identify which component or combination of components was responsible for the observed outcomes.

### 4.2. Future Research Directions

Future investigations should be registered prospectively before any children are enrolled. To detect genuinely small-to-moderate impacts and to determine whether results hold across preschools that vary in socioeconomic, cultural, geographic, and educational features, well-powered multisite trials are required.

An attention-matched active control group should be built into future trials. Suitable comparison tasks might involve adult-led storytelling, sedentary games, guided art sessions, or non-motor play, provided for the same length of time and with similar adult involvement. Such a design would make it easier to separate any motor play-specific benefits from influences due to novelty, attention, social contact, expectancy, and engagement in structured activities.

Outcome measurement also needs improvement through assessments that are independently validated, culturally appropriate, and developmentally appropriate. For replication, studies should describe the executive function and Go/No-Go procedures in enough detail, including the language of administration, practice and test trials, scoring criteria, assessor training, treatment of omissions and errors, and reliability estimates in the target population. In mathematics, outcomes should rely on broader standardized measures that contain untrained content, so that direct practice effects can be distinguished from transfer.

Future work should further examine baseline motor competence, socioeconomic indicators, habitual physical activity, sleep, nutrition, attendance, intervention fidelity, instructor training, teacher workload, implementation costs, safety, parent and school acceptability, and adverse events. Assessments at 3 and 6 months would also be useful for establishing whether immediate post-intervention differences endure and whether they relate to later school readiness or academic outcomes.

### 4.3. Practical Implications

The present study does not provide sufficient evidence for policy-level or routine implementation recommendations. It suggests only that structured motor play activities can be evaluated further as a possible preschool intervention model. Before broader implementation is considered, future research should determine whether effects exceed those observed with attention-matched non-motor activities and whether changes generalize to independently assessed developmental outcomes.

Recorded delivery indicators, including session completion, attendance, and video-reviewed adherence, describe what occurred in this single preschool and are distinct from a formal implementation evaluation covering costs, scalability, and acceptability. Implementation-related outcomes were not formally assessed in the present study. The research did not evaluate intervention costs, required equipment, instructor training time, teacher workload, attendance, fidelity, acceptability among parents or schools, or scalability across preschool settings. Accordingly, the program should be regarded as a candidate model for further evaluation rather than as a ready-to-implement educational intervention.

## 5. Conclusions

Over 12 weeks, structured motor play was associated with greater short-term improvement in Go/No-Go task performance and immediate affective valence than usual preschool activities. No between-group difference was observed in derived Stroop interference time. Although mathematics task scores increased more in the intervention group in the unadjusted analysis, this result was not maintained after false-discovery-rate correction, was marginal in the nonparametric sensitivity analysis, and was based on content directly practiced during the intervention.

These findings offer preliminary, task-specific estimates rather than definitive evidence that structured motor play improves generalized executive function, mathematics ability, school readiness, or sustained affective functioning. Larger prospectively registered and adequately powered multisite trials, using attention-matched active controls, validated assessments, implementation measures, and follow-up assessments, are needed to clarify the specificity, reproducibility, and durability of these findings.

## Figures and Tables

**Table 1:** Structure and content of the 12-week structured motor play program.

Weeks	Intervention Focus	Example of Motor Play Activities	Cognitive and Self-Regulatory Demands	Intervention-Aligned Conceptual Content
1–2	Familiarization with movement rules	Walking through obstacle paths, hopping between markers, rolling soft balls	Listening to instructions, following simple rules, attending to signals	Recognition of shapes and colors
3–4	Coordination and object manipulation	Throwing and catching soft balls, stepping through hoops, balance-beam walking	Response inhibition using stop–go signals, attention to changing instructions	Counting repetitions from 1 to 5
5–6	Locomotor control and spatial navigation	Crawling, jumping, zigzag running, navigating obstacle courses	Selecting a path, making simple decisions, monitoring movement rules	Spatial concepts, including inside/outside and near/far
7–8	Fine motor control and object manipulation	Shape-sorting stations, beanbag tossing, stacking blocks	Attention control, task switching, object selection	Shape identification, including circle and square
9–10	Cooperative movement challenges	Partner ball passing, group relays, synchronized jumping	Social regulation, turn taking, rule monitoring	Classification according to color or size
11	Integrated motor–cognitive tasks	Multi-station obstacle course involving jumping, balancing, and throwing	Remembering action sequences, following multi-step instructions	Sequencing and pattern recognition
12	Consolidation and game-based activities	Game-based circuits combining jumping, balancing, throwing, and object manipulation	Rapid decision-making, rule adherence, response inhibition	Counting scores and grouping objects

Note. The intervention combined gross-motor activity, fine motor activity, cognitive rules, social interaction, and simple mathematical content. Shape identification, counting from 1 to 5, and classification by color or size were explicitly practiced during the program and directly overlapped with the researcher-developed mathematics outcome. Therefore, mathematics task results should be interpreted as performance on intervention-aligned trained content, not as evidence of generalized mathematics ability, numeracy, or school readiness.

**Table 2:** Structure of each 45 min session.

Phase	Duration	Description
Warm-up	5–8 min	Light locomotor activities (walking, running, stretching) to prepare children for movement
Structured motor play activities	30 min	Structured motor play tasks combining gross and fine motor skills with cognitive rules
Game-based challenge	5–7 min	Cooperative or competitive game reinforcing movement skills
Cool-down	3–5 min	Breathing, stretching, and short reflection

Note. Sessions were scheduled twice weekly for 12 weeks and lasted approximately 45 min. Detailed information on session delivery, attendance, and fidelity is provided in Section 2.3 and Supplementary Table S4.

**Table 3:** Baseline demographic characteristics by group.

Characteristic	Experimental (*n* = 20)	Control (*n* = 20)
Boys, *n* (%)	8 (40.0%)	8 (40.0%)
Girls, *n* (%)	12 (60.0%)	12 (60.0%)
Mean age, years (SD)	4.08 (0.18)	4.04 (0.16)

Note. Values are presented descriptively. Given the small sample, non-significant baseline tests should not be interpreted as evidence that groups were equivalent on measured or unmeasured characteristics. No data were collected on baseline motor competence, socioeconomic circumstances, habitual physical activity, sleep, nutrition, or related developmental factors.

**Table 4:** Baseline, immediate post-intervention, and change score descriptive statistics.

Outcome	Group	Baseline, M (SD)	Immediate Post-Intervention, M (SD)	Change Score (*T*2 − *T*1), M (SD)
Stroop-derived interference time, s	Structured motor play	32.20 (3.30)	31.20 (5.28)	−1.00 (4.74)
	Usual preschool activities	32.85 (3.27)	32.00 (3.20)	−0.85 (1.42)
Go/No-Go commission errors	Structured motor play	2.15 (0.75)	1.60 (0.60)	−0.55 (0.51)
	Usual preschool activities	2.25 (0.55)	2.30 (0.57)	0.05 (0.22)
Brief intervention-aligned mathematics task, score 0–3	Structured motor play	1.90 (0.79)	2.40 (0.60)	0.50 (0.51)
	Usual preschool activities	1.60 (0.68)	1.80 (0.77)	0.20 (0.41)
Immediate affective valence, Feeling Scale	Structured motor play	2.40 (0.60)	3.85 (0.67)	1.45 (0.69)
	Usual preschool activities	3.65 (0.49)	3.70 (0.57)	0.05 (0.60)

Note. *N* = 40, with *n* = 20 per group. Change scores were calculated as immediate post-intervention minus baseline scores. Negative change scores for Stroop-derived interference time and Go/No-Go commission errors indicate lower scores at immediate post-intervention. Positive change scores for the intervention-aligned mathematics task and Feeling Scale indicate higher scores at immediate post-intervention. Stroop-derived interference time was calculated as Card 3 completion time minus Card 1 completion time. Card identities, administration language, and error-handling procedures are reported in Section 2.4.1. The mathematics task directly assessed shape identification, counting, and classification, all of which were practiced in the intervention. Therefore, it reflects intervention-aligned trained-task performance rather than generalized mathematics ability. The Feeling Scale is a single-item measure of immediate self-reported affective valence; Cronbach’s alpha is not applicable.

**Table 5:** Between-group differences in change scores following the motor play intervention.

Outcome	Structured Motor Play Group, Change M (SD)	Usual Preschool Activities Group, Change M (SD)	Welch’s t(df)	Unadjusted *p*	FDR-Adjusted q	Mann–Whitney U	Sensitivity *p*	Hedges’ g	95% Bootstrap CI
Stroop-derived interference time, s	−1.00 (4.74)	−0.85 (1.42)	−0.14 (22.41)	0.893	0.893	260.5	0.098	−0.04	−0.56 to 0.80
Go/No-Go commission errors	−0.55 (0.51)	0.05 (0.22)	−4.82 (26.03)	<0.001	<0.001	85.5	<0.001	−1.49	−2.34 to −0.99
Brief intervention-aligned mathematics task, score 0–3	0.50 (0.51)	0.20 (0.41)	2.04 (36.25)	0.048	0.065	260.0	0.051	0.63	0.00 to 1.45
Immediate affective valence, Feeling Scale	1.45 (0.69)	0.05 (0.60)	6.84 (37.41)	<0.001	<0.001	374.0	<0.001	2.12	1.55 to 3.23

Note. N = 40 (*n* = 20/group). Change scores = post minus baseline. Welch’s *t*-tests compared change scores without assuming equal variances; Mann–Whitney U served as a nonparametric sensitivity check. Hedges’ g values are exploratory standardized differences with 95% bootstrap CIs (2000 resamples, seed 20260917). Benjamini–Hochberg FDR correction was applied across the four comparisons. Negative values indicate larger reductions (Stroop, Go/No-Go) or increases (mathematics, Feeling Scale) in the structured motor play group; positive values indicate the reverse for mathematics and Feeling Scale. The mathematics result did not survive FDR correction (q = 0.065) and was marginal on Mann–Whitney (*p* = 0.051), so it is regarded as inconclusive given the restricted 0–3 range and content overlap with the intervention. For Feeling Scale, a baseline-adjusted sensitivity analysis (Section 3.2) corroborated the change score result; the homogeneity-of-slopes assumption held only for this outcome (interaction *p* = 0.883).

## Data Availability

The original contributions presented in the study are included in the article, further inquiries can be directed to the correspondent author.

## Supplementary Materials

The following supporting information can be downloaded at: https://www.mdpi.com/article/10.3390/children13101377/s1, Table S1: Administration, Scoring, and Available Measurement Information for Study Outcomes; Table S2: Score Frequencies for the Brief Intervention-Aligned Mathematics Task by Group and Assessment Occasion; Table S3: Sensitivity Analyses for the Brief Intervention-Aligned Mathematics Task; Table S4: Intervention Delivery and Available Fidelity Indicators; Table S5: Transparency Classification of Outcomes and Analyses.
