# Supplementary material for: Structured Motor Play and Preschool Task Performance: An Exploratory Single-Site Randomized Pilot Study in Tunisian Children
Source: Children (Basel). 2026 Oct 10;13(10):1377. doi: 10.3390/children13101377 (PMC13703156; doi:10.3390/children13101377)
Supplement: Supplementary file 1 [file children-13-01377-s001.zip › children-4553717-supplementary.pdf]

Supplementary Table S1. Administration, Scoring, and Available Measurement Information for Study Outcomes

Outcome	Administration information reported in the study	Primary score and direction	Scoring rule reported	Reliability/validity information available
**Stroop task**	Victoria Stroop Color–Word Test; administered individually in Arabic by trained research personnel	Derived interference time (s); lower = lower interference time	Card 3 completion time − Card 1 completion time, calculated from full completion times for all 40 children (no incomplete administrations occurred)	Not independently validated in Tunisian preschool children
**Go/No-Go task**	Simplified tap/double-tap paradigm adapted from the Frontal Assessment Battery; 2 practice trials followed by 6 test trials (3 Go, 3 No-Go, fixed alternating order); administered individually in Arabic	Number of commission errors, 0–3; lower = fewer errors	A commission error is a response during a No-Go (double-tap) trial; omissions and unclear responses scored as correct inhibition	Not independently validated in Tunisian preschool children; Frontal Assessment Battery citation is task provenance only, not evidence of psychometric validity in this population
**Intervention-aligned mathematics task**	Individually administered at baseline and post-intervention in a quiet preschool area; standardized instructions; identical materials at both occasions; ~3–5 min per child	Total score 0–3; higher = more correct responses	One point per correct response on 3 items (shape identification, counting 1–5, classification); instructions repeatable once, no feedback	Six-child feasibility pilot: alpha = .85, test–retest r = .72 (unstable, not formal validation)
**Feeling Scale**	Individually administered at baseline and post-intervention in a quiet preschool area; visual display with numbers and facial symbols;	Single rating, −5 to +5; higher = more positive immediate valence	Child points to number/symbol; instructions repeatable once, no feedback	Six-child pilot supported feasibility/comprehension only

	standardized Tunisian Arabic prompt			

Supplementary Table S2. Score Frequencies for the Brief Intervention-Aligned Mathematics Task by Group and Assessment Occasion

Score	Motor play, baseline n (%)	Motor play, post n (%)	Usual activities, baseline n (%)	Usual activities, post n (%)
**0**	0 (0.0)	0 (0.0)	0 (0.0)	0 (0.0)
**1**	7 (35.0)	1 (5.0)	10 (50.0)	8 (40.0)
**2**	8 (40.0)	10 (50.0)	8 (40.0)	8 (40.0)
**3**	5 (25.0)	9 (45.0)	2 (10.0)	4 (20.0)
**Total**	20 (100.0)	20 (100.0)	20 (100.0)	20 (100.0)

Note. No child scored 0 at either occasion. These frequencies are computed directly from the participant-level dataset and illustrate the bounded, discrete nature of the outcome. They should not be interpreted as evidence of generalized mathematics learning given the direct overlap with intervention content.

Supplementary Table S3. Sensitivity Analyses for the Brief Intervention-Aligned Mathematics Task

Analysis	Result	Interpretation
**Welch's t-test on change scores**	$t(36.25) = 2.04$, $p = .048$	Nominally significant, unadjusted
**Benjamini–Hochberg FDR adjustment (4 outcomes)**	$q = .065$	Not significant after adjustment
**Mann–Whitney U**	$U = 260.0$, $p = .051$	Marginal, not significant at $\alpha = .05$
**Exact two-sided permutation test**	Observed difference = 0.30; $p = .096$	Not significant
**Hedges' g**	$g = 0.63$, 95% CI [0.00, 1.45]	Exploratory, wide uncertainty

Note. Inconsistency across these four analyses indicates the mathematics result is not robust and should be reported as inconclusive exploratory evidence of trained-task performance only.

Supplementary Table S4. Intervention Delivery and Available Fidelity Indicators

Indicator	Information reported in the manuscript
**Planned duration**	12 weeks, two ~45-minute sessions/week (24 sessions total)

**Sessions delivered**	24 of 24
**Attendance**	Mean 93% ; range 85%–100%
**Fidelity method**	Video review by trained research personnel
**Fidelity result**	Mean adherence ≈ 95%; no major deviations identified
**Instructors**	Trained physical-education specialists (number and qualifications not specified)
**Setting**	One preschool, Manouba, Tunisia
**Implementation cost, teacher workload, parent/school acceptability, scalability**	Not formally assessed
**Fidelity-assessment scope**	Video review by a single trained rater against the intervention protocol; this constitutes a descriptive adherence estimate rather than a multi-rater formal fidelity assessment.

Note. These indicators describe delivery in one preschool only and cannot support feasibility, cost-effectiveness, or scalability claims.

Supplementary Table S5. Transparency Classification of Outcomes and Analyses

Outcome/analysis	Classification	Basis
**Derived Stroop interference time**	Exploratory outcome	Retrospective registration; no prospective protocol
**Go/No-Go commission errors**	Exploratory outcome	Retrospective registration; no prospective protocol
**Mathematics task**	Exploratory outcome	Retrospective registration; direct content overlap with intervention
**Feeling Scale**	Exploratory outcome	Retrospective registration; single-item measure
**Welch t-tests**	Exploratory analysis	No prospective statistical analysis plan
**Mann–Whitney U**	Exploratory sensitivity analysis	Robustness check for bounded/discrete/non-normal outcomes
**Benjamini–Hochberg FDR**	Post hoc multiplicity adjustment	Applied across 4 exploratory comparisons
**Exact permutation test (mathematics)**	Post hoc sensitivity analysis	Added due to bounded, discrete outcome
**Hedges' g with bootstrap CI**	Exploratory effect-size estimate	Small-sample estimate
